# Supplementary material for: Distinct retrograde microtubule motor sets drive early and late endosome transport
Source: EMBO J. 2020 Nov 20;39(24):e103661. doi: 10.15252/embj.2019103661 (PMC7737607; doi:10.15252/embj.2019103661)
Supplement: Supplementary file 1 — Expanded View Figures PDF [file EMBJ-39-e103661-s001.pdf]

Expanded View Figures

Figure EV1. STIM1 drives late endosome retrograde transport without affecting the assembly of the dynein/dynactin complex, showing an additional function other than ER calcium sensor.

A Representative Western blot analysis of the endogenous STIM1 in ECs silenced with a control shRNA (shCTL) or three different tested shRNAs targeting STIM1 (shSTIM1) (left) and its quantification by normalized densitometry (right). Results are the average ± SD of three independent assays and were analyzed by a parametric two-tailed analysis of variance (ANOVA) with Bonferroni *post hoc* analysis. ANOVA $P \leq 0.001$; Bonferroni for shCTL and sh#490 $P \leq 0.001$***, for shCTL and sh#718 $P \leq 0.01$** and for shCTL and sh#780 $P \leq 0.01$**. In this manuscript, only the #780 was used.

B Representative Western blot analysis of endogenous light-intermediate chain (LIC) of cytoplasmic dynein 1 co-immunoprecipitated with p150Glued in shCTL or shSTIM1 ECs (left) and its quantification by normalized densitometry (right). Results are the average ± SD of three independent assays. ShCTL value of each biological replicate was normalized on itself and so shSTIM1 experimental value. Results were analyzed by a two-tailed heteroscedastic Student's *t*-test, $P > 0.05$ not significant (ns).

C Representative Western blot analysis of GFP-STIM1 WT, ΔCC1, ΔCC2, or ΔCC3 co-immunoprecipitated with mCherry-p150Glued WT in cotransfected HEK 293T cells. Negative control (CTL) was performed incubating cell lysate from HEK 293T cotransfected with an empty GFP vector together with mCherry-P150Glued WT with pre-cleared protein A or G-Sepharose and the rabbit GFP antibody. Right, its quantification by normalized densitometry. Results are the average ± SD of three independent assays. The value of p150Glued co-immunoprecipitated with GFP-STIM1 WT from each biological replicate was normalized on itself and so those immunoprecipitated with GFP-STIM1 ΔCC1, ΔCC2, or ΔCC3. Results were analyzed by a parametric two-tailed analysis of variance (ANOVA) with Bonferroni *post hoc* analysis. ANOVA $P \leq 0.01$**; Bonferroni for STIM1 WT and ΔCC1 $P > 0.05$ not significant (ns), for STIM1 WT and ΔCC2 $P > 0.05$ not significant (ns) and for STIM1 WT and ΔCC3 $P > 0.05$ not significant.

D Representative Western blot analysis of the endogenous STIM1 in ECs silenced with a control siRNA (siCTL) or one targeting STIM1 (siSTIM1) (left) and its quantification by normalized densitometry (right). Results are the average ± SD of three independent assays. Results were analyzed by a two-tailed heteroscedastic Student's *t*-test, $P \leq 0.001$***.

E Confocal microscopy images of untreated (UT) or treated with Thapsigargin (TG) ECs and stained for endogenous LAMP-1 (in green) to visualize LEs and DAPI (in blue) to highlight the nucleus. The yellow line is drawn to define cell periphery. Scale bar = 20 μm. On the right, inset panels to highlight respective perinuclear and peripheral area of the cell. Scale bar = 5 μm.

F Distribution of distance to nucleus, normalized on cell size, quantified by image (as in D) segmentation (see Materials and Methods, Confocal microscopy and early/late quantification) of LAMP-1^+ endosomes. Results are from three independent experiments for a total of 441 late endosomes in 13 UT cells (34 ± 4 endosomes per cell) and 519 late endosomes in 18 TG cells (29 ± 3 endosomes per cell) and analyzed by a two-tailed heteroscedastic Student's *t*-test, $P \leq 0.001$***.

Source data are available online for this figure.

 © 2020 The Authors

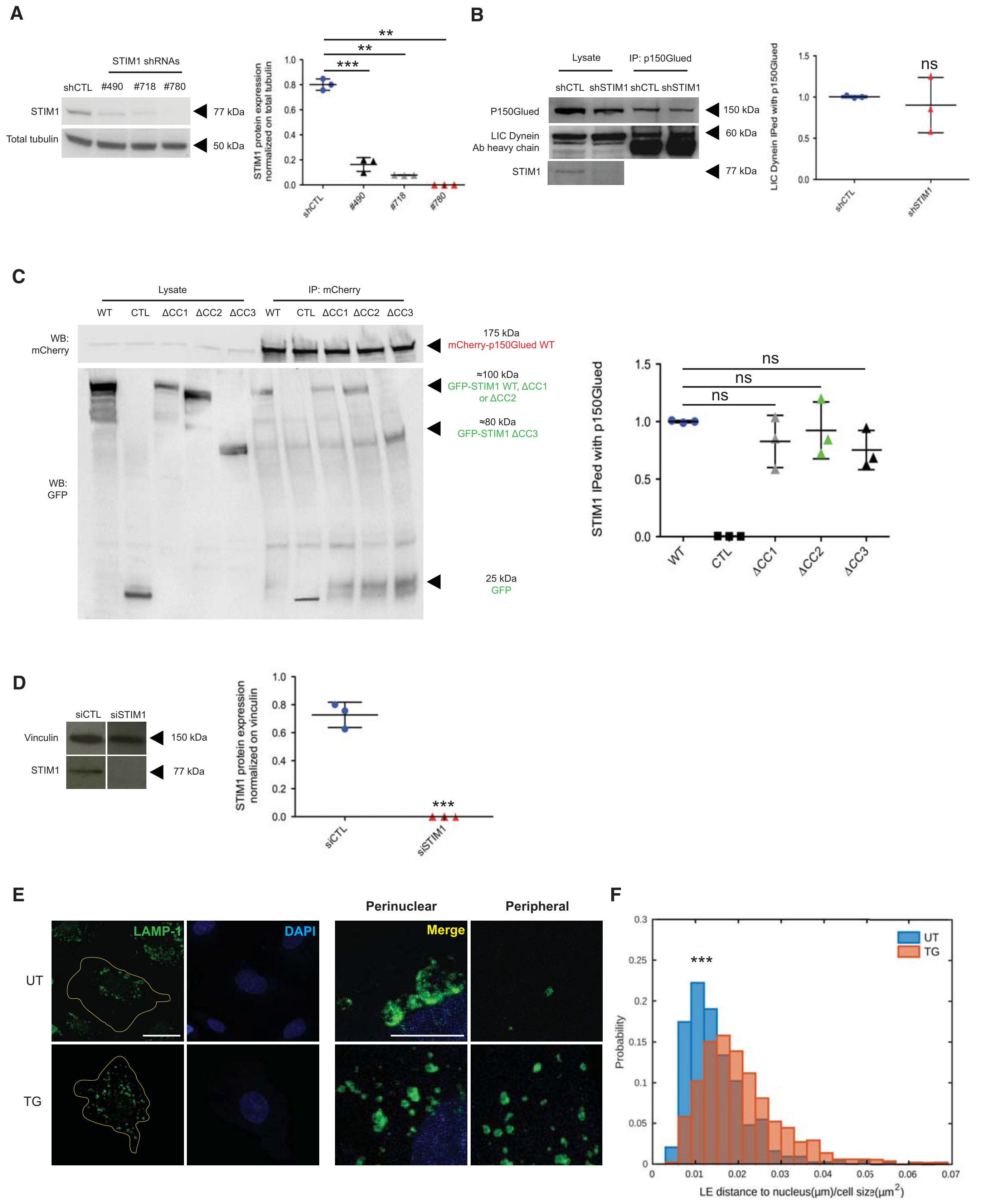
A
STIM1 shRNAs
shCTL #490 #718 #780
STIM1
Total tubulin
77 kDa
50 kDa
STIM1 protein expression normalized on total tubulin
**
**
***
shCTL #490 #718 #780
B
Lysate
IP: p150Glued
shCTL shSTIM1 shCTL shSTIM1
P150Glued
LIC Dynein Ab heavy chain
STIM1
150 kDa
60 kDa
77 kDa
LIC Dynein IPed with p150Glued
ns
shCTL shSTIM1
C
Lysate
IP: mCherry
WT CTL ΔCC1 ΔCC2 ΔCC3 WT CTL ΔCC1 ΔCC2 ΔCC3
WB: mCherry
WB: GFP
175 kDa
mCherry-p150Glued WT
≈100 kDa
GFP-STIM1 WT, ΔCC1 or ΔCC2
≈80 kDa
GFP-STIM1 ΔCC3
25 kDa
GFP
STIM1 IPed with p150Glued
ns
ns
ns
WT CTL ΔCC1 ΔCC2 ΔCC3
D
siCTL siSTIM1
Vinculin
STIM1
150 kDa
77 kDa
STIM1 protein expression normalized on vinculin
***
siCTL siSTIM1
E
Perinuclear
Peripheral
LAMP-1
DAPI
Merge
UT
TG
F
UT
TG
***
Probability
LE distance to nucleus(µm)/cell size(µm2)

Figure EV1.

Figure EV2. Dynein and KIFC1 differentially control early and late endosome retrograde transport in human cancer cells and fibroblasts, counteracting KIF5B in early endosome motion along MTs.

A Colocalization analysis of EEA-1$^+$ EEs with LAMP-1$^+$ LEs in siCTL and siSTIM1 ECs. Results are the average ± SEM of three independent experiments for a total of 90 cells (30 cell for experiment) and analyzed by a two-tailed heteroscedastic Student's *t*-test, $P > 0.05$ not significant (ns).

B Confocal microscopy images of untreated (UT) or treated with Ciliobrevin D (CilioD) or AZ82 Hs746T carcinoma cells and stained for endogenous EEA-1 (in green) and LAMP-1 (in red) to visualize EEs and LEs, respectively, and Draq5 (in blue) to highlight the nucleus. The yellow line is drawn to define cell periphery. Scale bar = 20 μm. On the right, inset panels to highlight respective perinuclear and peripheral area of the cell. Scale bar = 5 μm.

C Confocal microscopy images of untreated (UT) or treated with Ciliobrevin D (CilioD) or AZ82 MRC5 fibroblasts and stained for endogenous EEA-1 (in green) and LAMP-1 (in red) to visualize EEs and LEs, respectively, and Draq5 (in blue) to highlight the nucleus. The yellow line is drawn to define cell periphery. Scale bar = 20 μm. On the right, inset panels to highlight respective perinuclear and peripheral area of the cell. Scale bar = 5 μm.

D Distribution of distance to nucleus, normalized on cell size, quantified by image (as in Fig 5C) segmentation (see Materials and Methods, Confocal microscopy and early/late quantification) of LAMP-1$^+$ endosomes in siCTL and siKIFC1 ECs. Results are from three independent experiments for a total of 906 late endosomes in 19 siCTL cells (48 ± 6 endosomes per cell) and 417 late endosomes in 20 siKIFC1 cells (21 ± 2 endosomes per cell) and analyzed by a two-tailed heteroscedastic Student's *t*-test, $P > 0.05$ not significant (ns).

E Confocal microscopy images of siCTL ECs and cells silenced with a siRNA targeting KIF5B (siKIF5B) and stained for endogenous EEA-1 (in green) to visualize EEs and DAPI (in blue) to highlight the nucleus. Top part shows Western blot analysis of KIF5B silencing in ECs. In the bottom images, the yellow line is drawn to define cell periphery. Scale bar = 20 μm. On the right, inset panels to highlight respective perinuclear and peripheral area of the cell. Scale bar = 5 μm.

Source data are available online for this figure.

 © 2020 The Authors

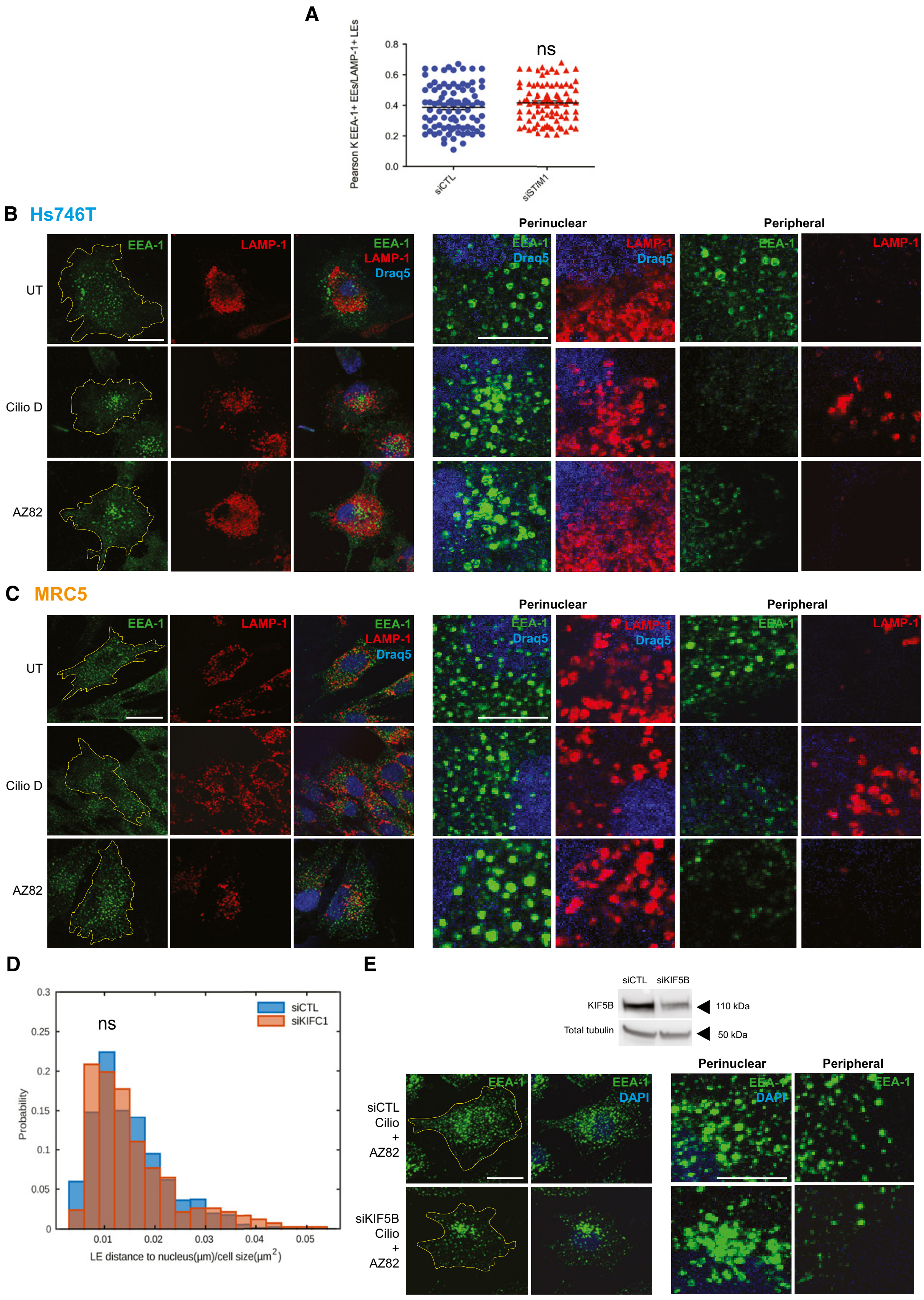
A
Pearson K EEA-1+ EEs/LAMP-1+ LEs
ns
siCTL
siSTIM1
B Hs746T
Perinuclear
Peripheral
EEA-1
LAMP-1
Draq5
UT
Cilio D
AZ82
C MRC5
D
ns
siCTL
siKIFC1
Probability
LE distance to nucleus(μm)/cell size(μm^2)
E
siCTL
siKIF5B
KIF5B
Total tubulin
110 kDa
50 kDa
siCTL Cilio + AZ82
siKIF5B Cilio + AZ82
DAPI

Figure EV2.

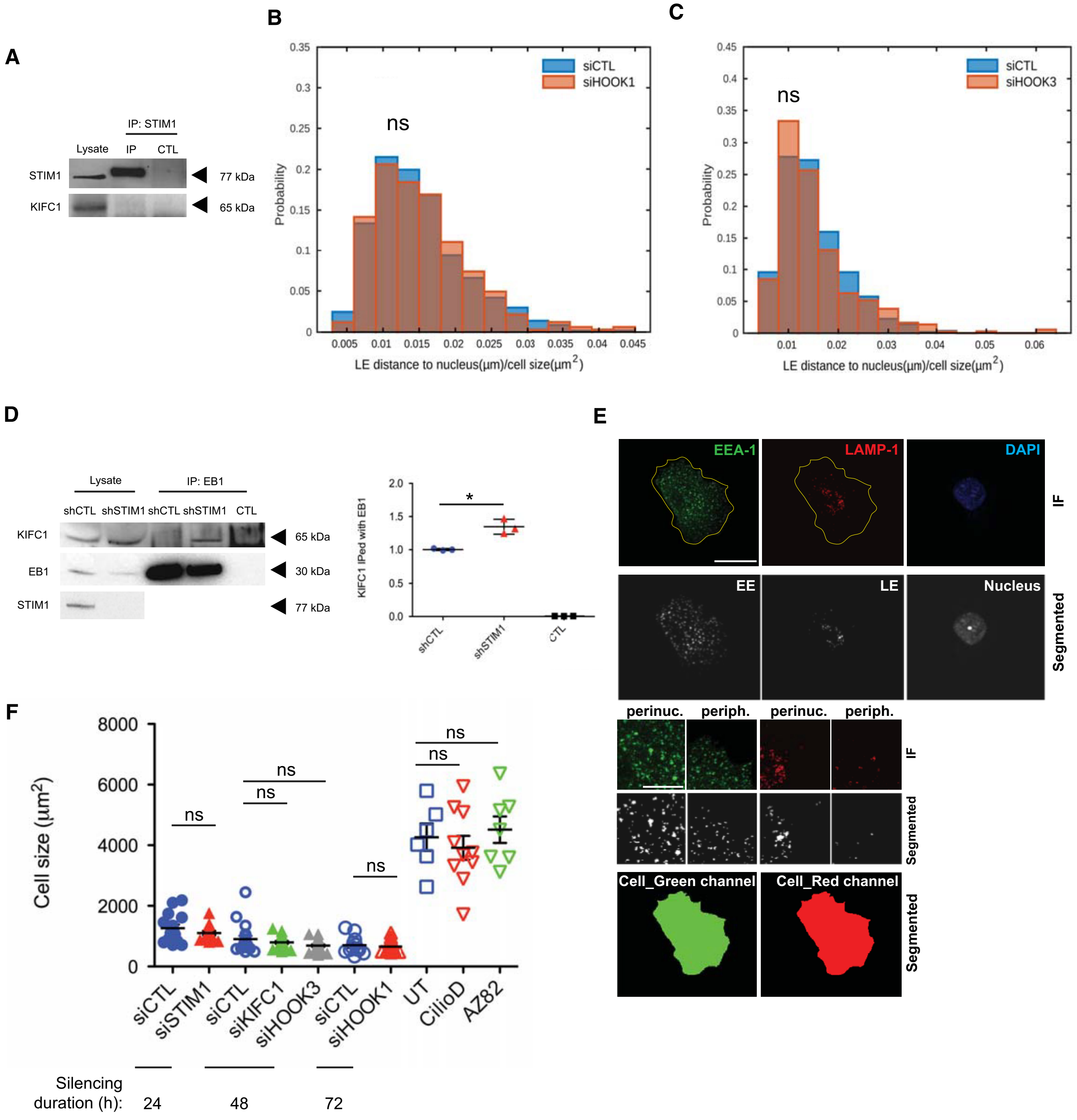

Figure EV3.

 © 2020 The Authors

Figure EV3. The STIM1/dynein and KIFC1 motor sets compete for the binding to the MT plus end protein EB1, exploiting specific HOOK adaptors to regulate early endosome retrograde transport.

A Representative of three Western blot analysis of endogenous KIFC1 immunoprecipitated with STIM1 in wild-type ECs. Negative control (CTL) was performed incubating cell lysate with protein A- or G-Sepharose and empty rabbit IgG.

B Distribution of distance to nucleus, normalized on cell size (shown in Fig 7E), quantified by image (as in Fig 7D) segmentation (see Materials and Methods, Confocal microscopy and early/late quantification) of LAMP-1^{+} endosomes. Results are from three independent experiments for a total of 576 late endosomes in 12 siCTL cells (48 ± 6 endosomes per cell) and 325 late endosomes in 21 siHOOK1 cells (15 ± 1 endosomes per cell) and analyzed by a two-tailed heteroscedastic Student's *t*-test, $P > 0.05$ not significant (ns).

C Distribution of distance to nucleus, normalized on cell size (shown in Fig 7F), quantified by image (as in Fig 7D) segmentation (see Materials and Methods, Confocal microscopy and early/late quantification) of LAMP-1^{+} endosomes. Results are from three independent experiments for a total of 576 late endosomes in 12 siCTL cells (48 ± 6 endosomes per cell) and 366 late endosomes in 22 siHOOK3 cells (16 ± 1 endosomes per cell) and analyzed by a two-tailed heteroscedastic Student's *t*-test, $P > 0.05$ not significant (ns).

D Representative Western blot analysis of the endogenous KIFC1 co-immunoprecipitated with EB1 in shCTL or shSTIM1 ECs (left) and its quantification by normalized densitometry (right). Negative control (CTL) was performed incubating cell lysate with protein A- or G-Sepharose and empty mouse IgG. Results are the average ± SD of three independent assays. shCTL value of each biological replicate was normalized on itself and so shSTIM1 experimental value. Results were analyzed by a parametric two-tailed analysis of variance (ANOVA) with Bonferroni *post hoc* analysis. ANOVA $P \leq 0.001$; Bonferroni for shCTL and shSTIM1 $P \leq 0.05$*.

E Representative image segmentation, generated by our automated quantification method, used to quantify fluorescent staining of EEs, LEs, nucleus, and cell area. The white dot into the nucleus segmentation represents its centroid (see Materials and Methods, Confocal microscopy and early/late quantification). The yellow line is drawn to define cell periphery. Scale bar = 20 μm. Lower panels show insets to highlight respective perinuclear and peripheral area of the cell. Scale bar = 5 μm.

F Average cell size after silencing and treatment experiments not introducing cell size bias. Results are the average ± SEM of three independent experiments for a total of 15 siCTL (24 or 48 or 72 h silencing duration), 13 siSTIM1 (24 h silencing duration), 19 siCTL (48 h silencing duration), 20 siKIFC1 (48 h silencing duration), 22 siHOOK3 (48 h silencing duration), 12 siCTL (72 h silencing duration), 21 siHOOK1 (72 h silencing duration), six untreated (UT), 10 CilioD, and seven AZ82-treated cells and analyzed by parametric two-tailed analysis of variance (ANOVA) with Bonferroni *post hoc* analysis. ANOVA $P > 0.05$ not significant (ns); Bonferroni for siSTIM1 and its 24 h siCTL $P > 0.05$ not significant (ns), for siKIFC1 or siHOOK3 and their 48 h siCTL $P > 0.05$ not significant (ns), for siHOOK3 and its 72 h siCTL $P > 0.05$ not significant (ns), and for CilioD or AZ82 with UT $P > 0.05$ not significant.

Source data are available online for this figure.

© 2020 The Authors
