# Supplementary material for: Distinct retrograde microtubule motor sets drive early and late endosome transport
Source: EMBO J. 2020 Nov 20;39(24):e103661. doi: 10.15252/embj.2019103661 (PMC7737607; doi:10.15252/embj.2019103661)
Supplement: Supplementary file 6 — Movie EV5 [file EMBJ-39-e103661-s006.zip › Movie EV5.docx]

**Movie EV5.** Movie performed by confocal microscopy in AZ82-treated ECs, transduced with GFP-Rab5. Scale bar = 20 μm. Each frame was taken every 0,5 second.
