# Supplementary material for: Distinct retrograde microtubule motor sets drive early and late endosome transport
Source: EMBO J. 2020 Nov 20;39(24):e103661. doi: 10.15252/embj.2019103661 (PMC7737607; doi:10.15252/embj.2019103661)
Supplement: Supplementary file 10 — Source Data for Figure 1 [file EMBJ-39-e103661-s008.zip › Source_Data_Figure_1-fig/Figure 1_Source data.pdf]

Figure 1A

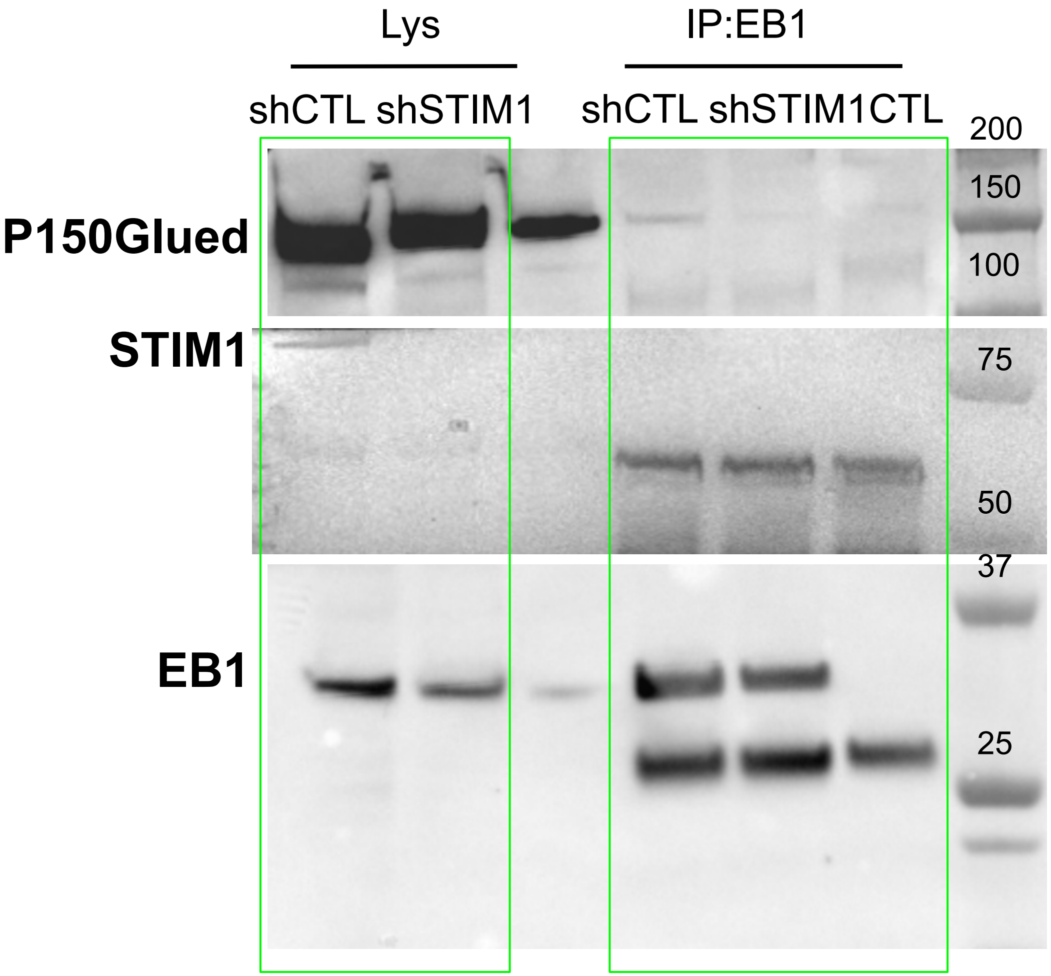

Highlighted in green what it has been cropped and shown in the relative Figure

Figure 1B

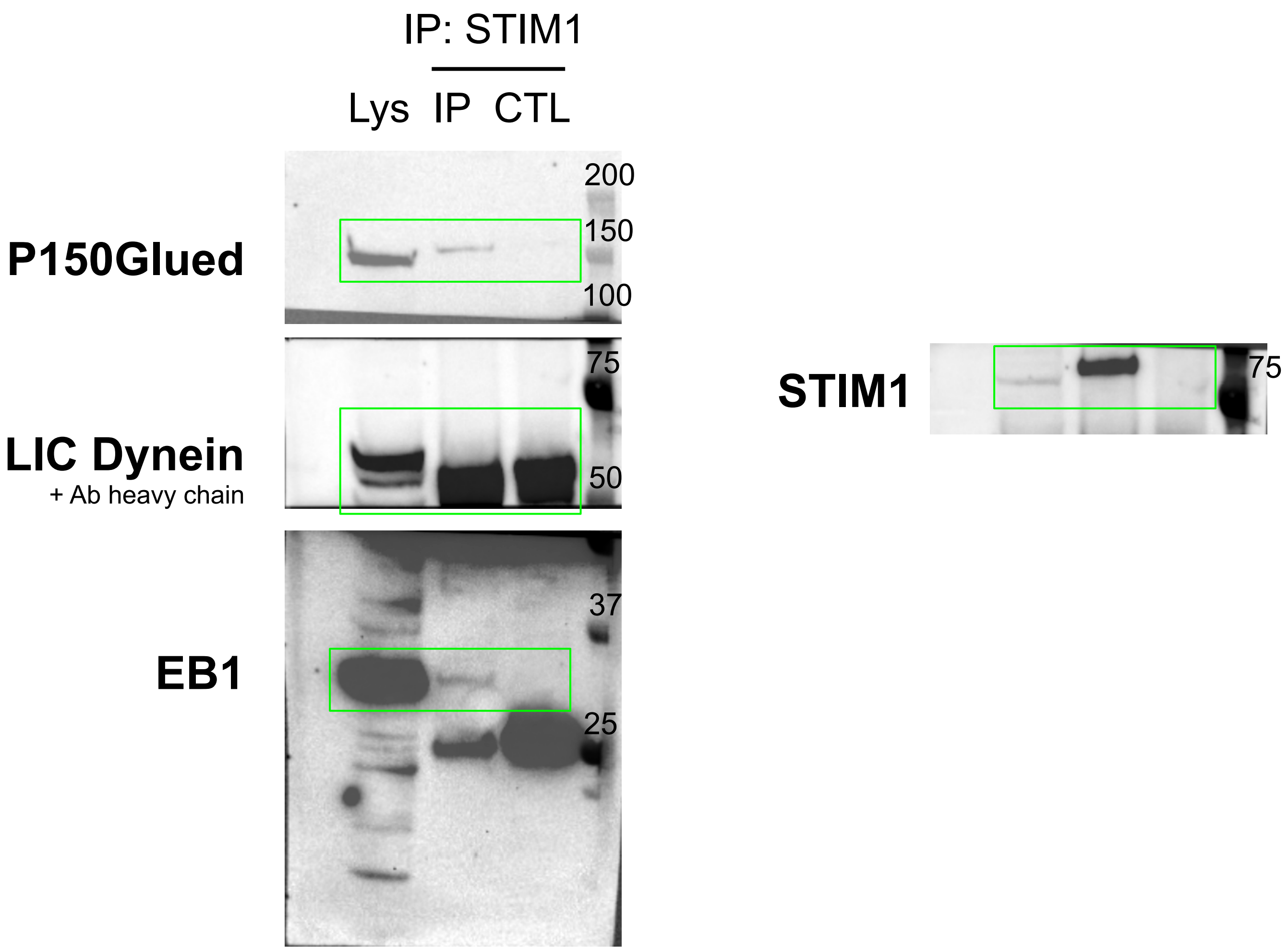
IP: STIM1
Lys
IP
CTL
P150Glued
200
150
100
LIC Dynein
+ Ab heavy chain
75
50
EB1
37
25
STIM1
75

Figure 1D

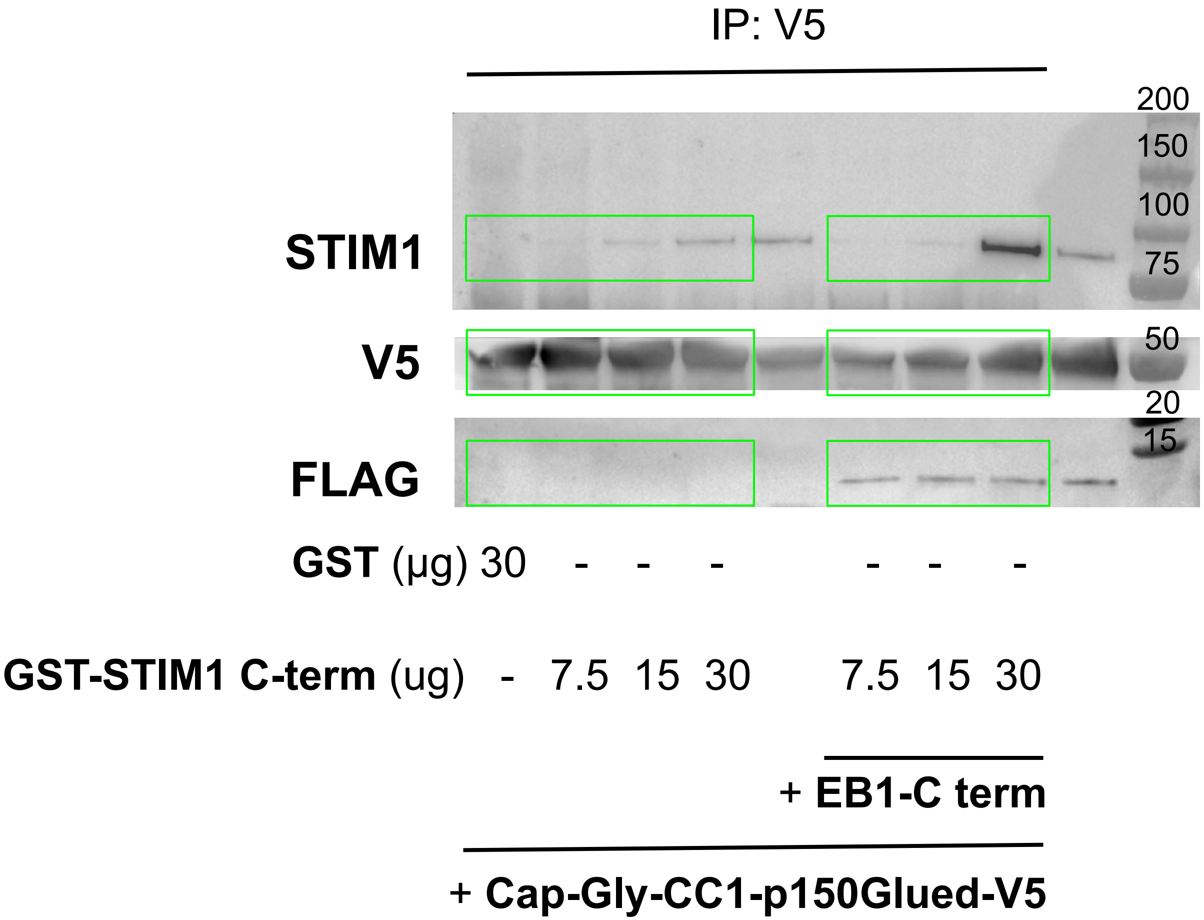
IP: V5
200
150
100
75
50
20
15
STIM1
V5
FLAG
GST (µg) 30 - - - - - -
GST-STIM1 C-term (ug) - 7.5 15 30 7.5 15 30
+ EB1-C term
+ Cap-Gly-CC1-p150Glued-V5

Figure 1E

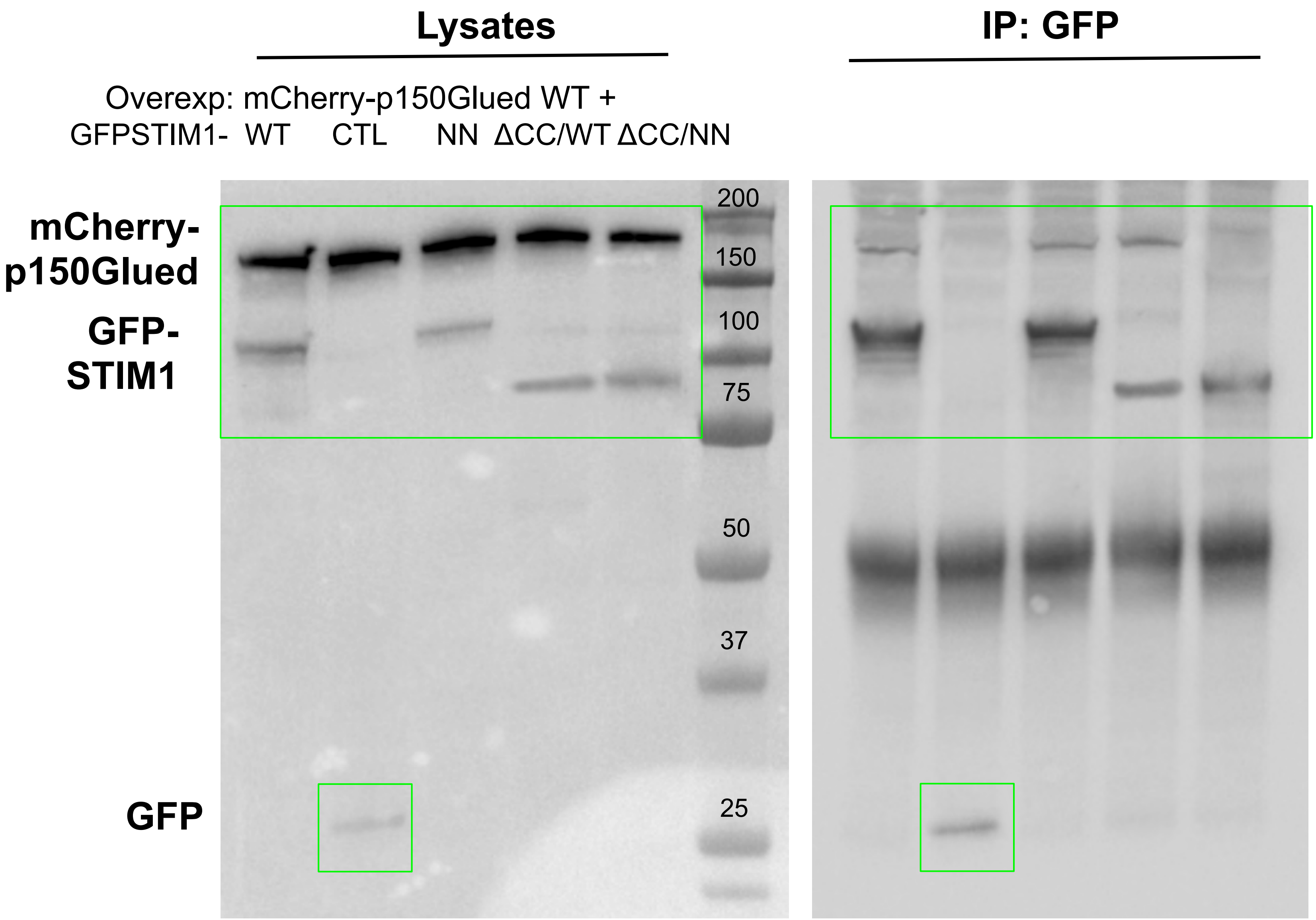

Anti-mCherry used to detect p150-Glued WT; antiGFP used to detect GFP-tagged STIM1 constructs

Figure 1F

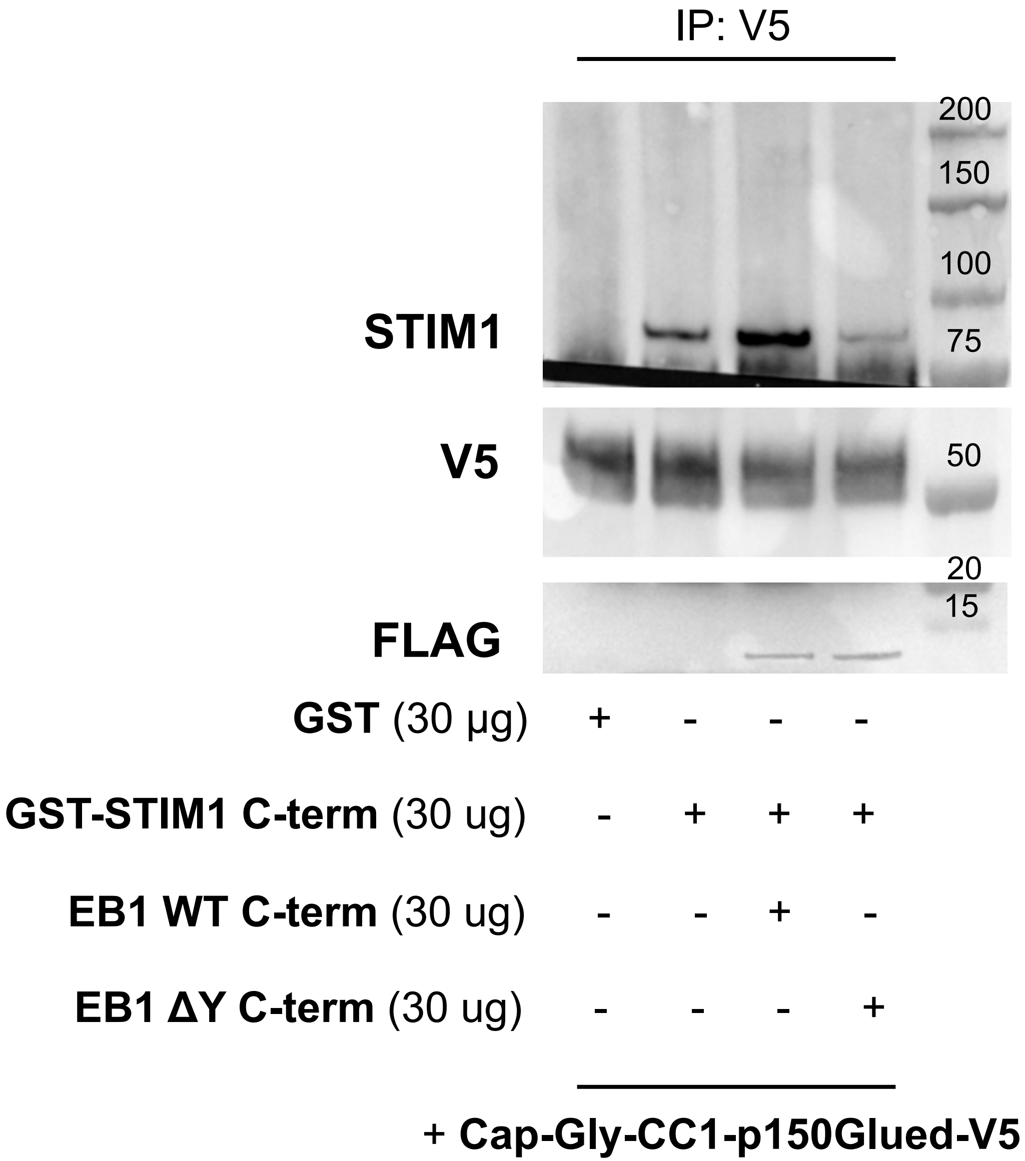
IP: V5
200
150
100
75
50
20
15
STIM1
V5
FLAG
GST (30 µg) + - - -
GST-STIM1 C-term (30 ug) - + + +
EB1 WT C-term (30 ug) - - + -
EB1 ΔY C-term (30 ug) - - - +
+ Cap-Gly-CC1-p150Glued-V5
