# Supplementary figures and images for: Distinct retrograde microtubule motor sets drive early and late endosome transport
Source: EMBO J. 2020 Nov 20;39(24):e103661. doi: 10.15252/embj.2019103661 (PMC7737607; doi:10.15252/embj.2019103661)

**Figure 5B**

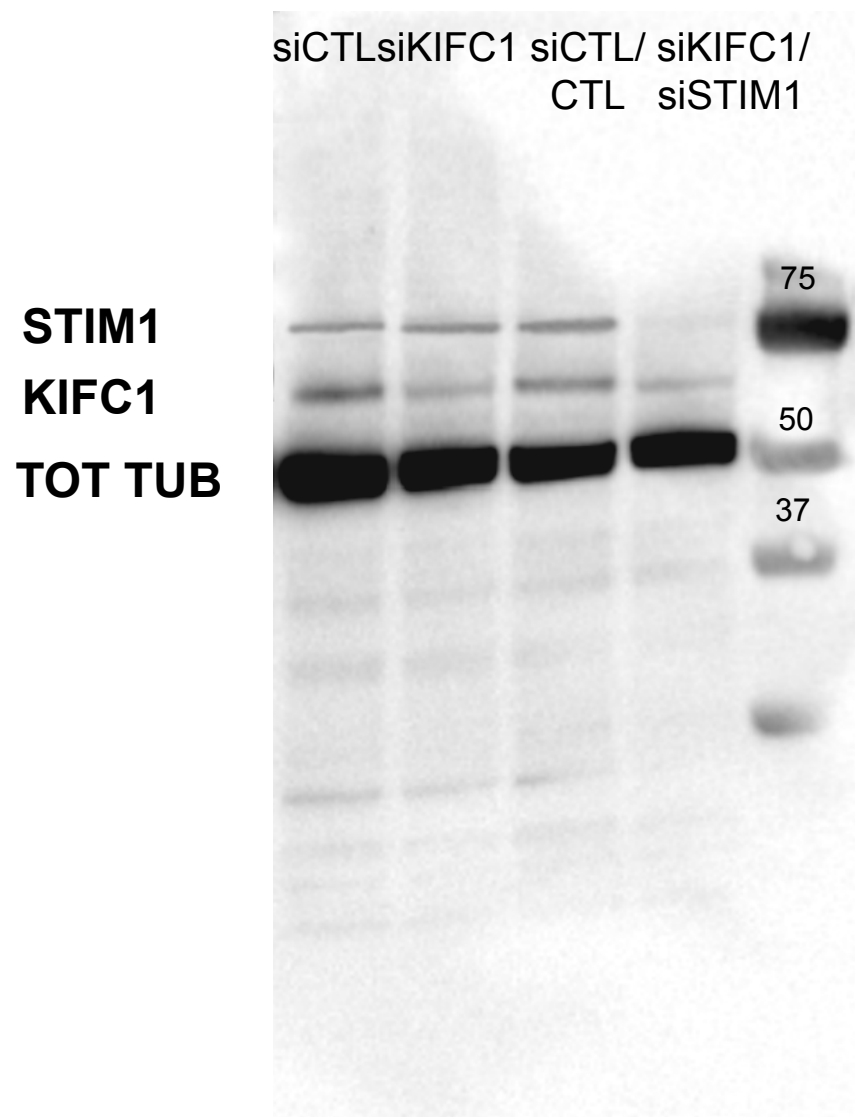

**siRNA against KIFC1 mRNA was used after 48h of cell culture**

Supplement: Supplementary file 14 — Source Data for Figure 5 [file EMBJ-39-e103661-s012.zip › Source_Data_Figure_5-fig/Figure 5_Source data.pdf]

Figure 8C

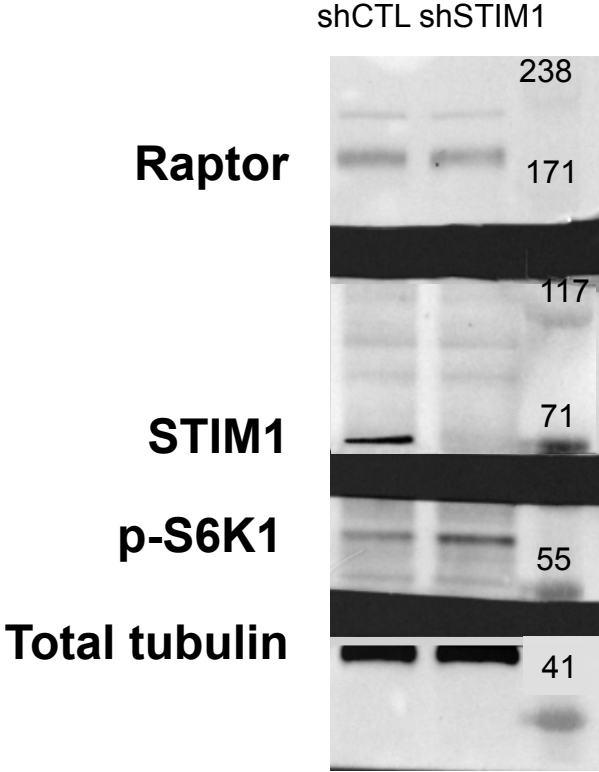

Gel run with Tris Acetate  
buffer to detect high  
molecular weight proteins

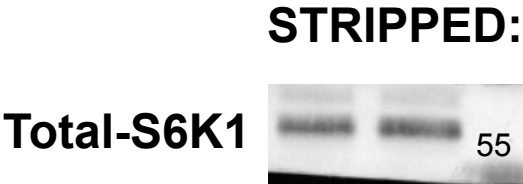

Figure 8D

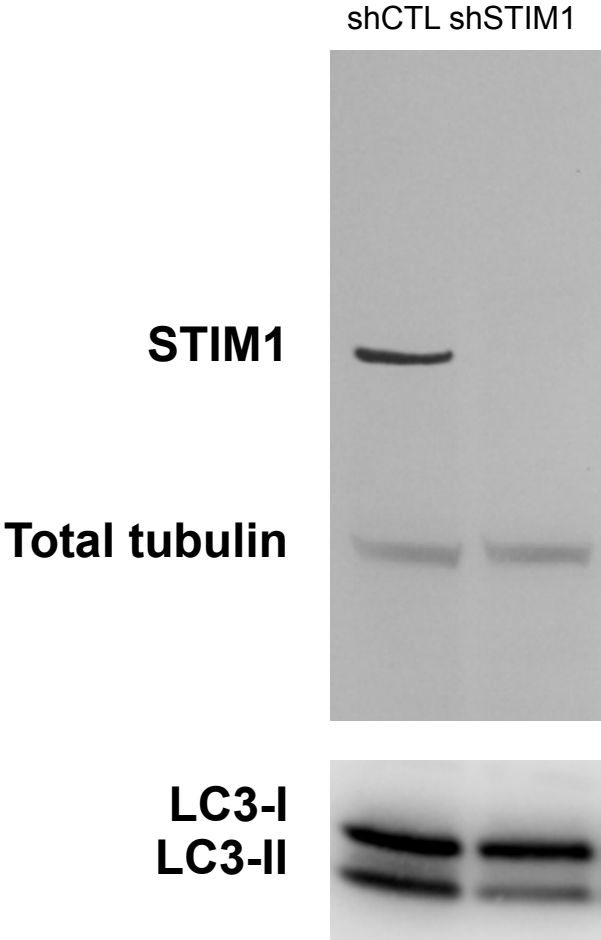

Supplement: Supplementary file 17 — Source Data for Figure 8 [file EMBJ-39-e103661-s015.zip › Source_Data_Figure_8-fig/Figure 8_Source data.pdf]
