# Supplementary material for: Distinct retrograde microtubule motor sets drive early and late endosome transport
Source: EMBO J. 2020 Nov 20;39(24):e103661. doi: 10.15252/embj.2019103661 (PMC7737607; doi:10.15252/embj.2019103661)
Supplement: Supplementary file 16 — Source Data for Figure 7 [file EMBJ-39-e103661-s014.zip › Source_Data_Figure_7-fig/Figure 7_Source data.pdf]

Figure 7A

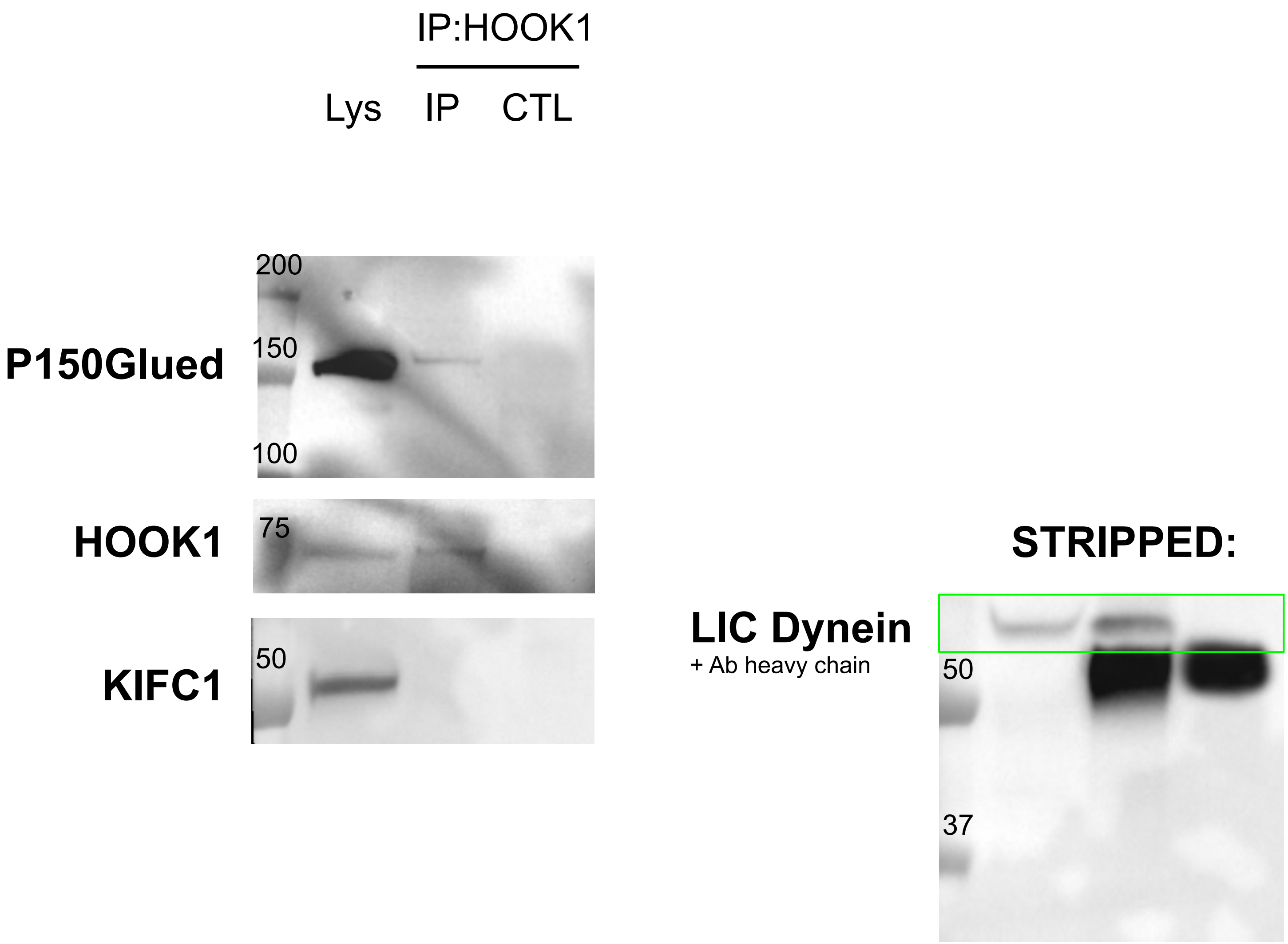
IP:HOOK1
Lys
IP
CTL
P150Glued
200
150
100
HOOK1
75
KIFC1
50
STRIPPED:
LIC Dynein
+ Ab heavy chain
50
37

Figure 7B

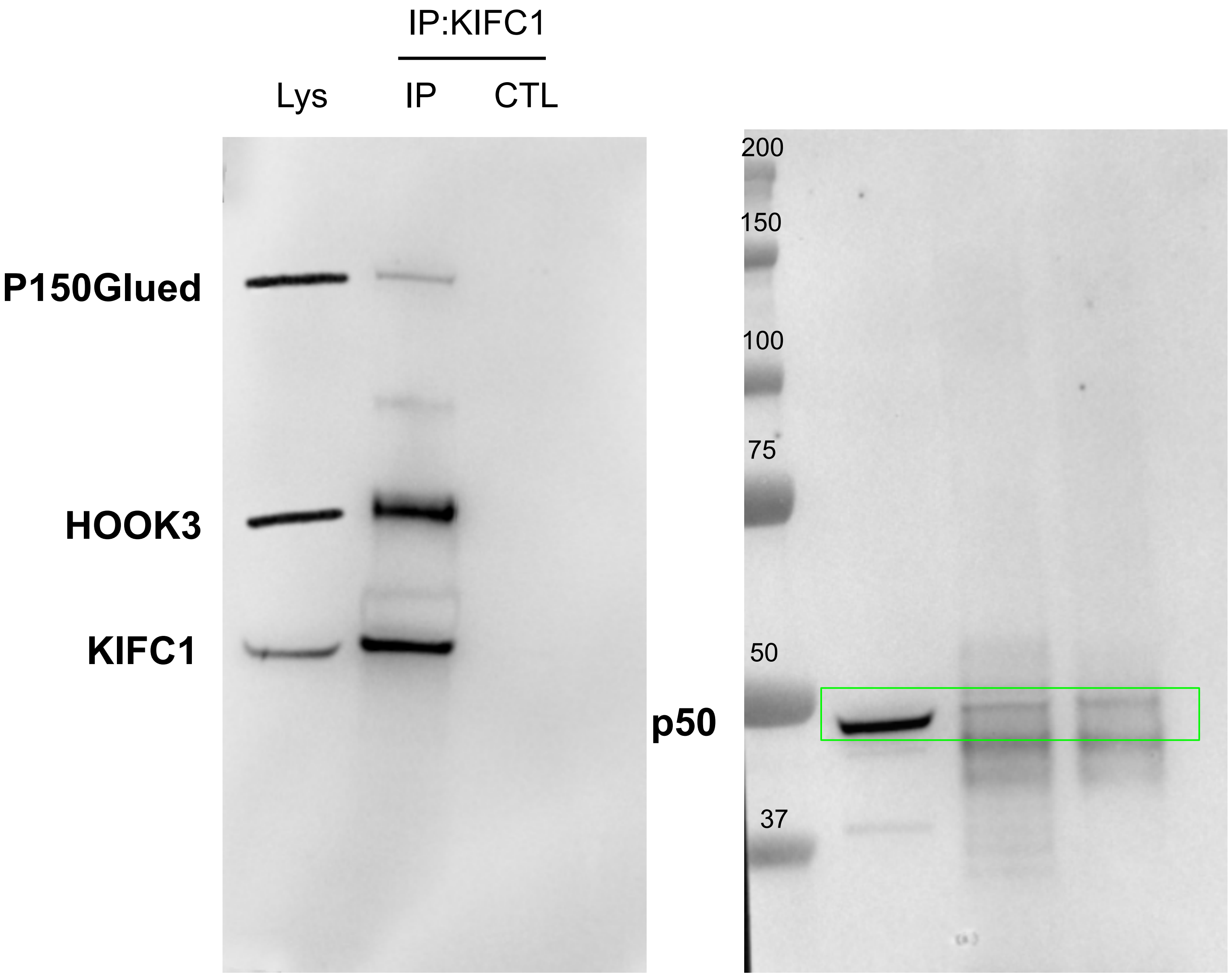
IP:KIFC1
Lys
IP
CTL
P150Glued
HOOK3
KIFC1
200
150
100
75
50
37
p50

Figure 7C

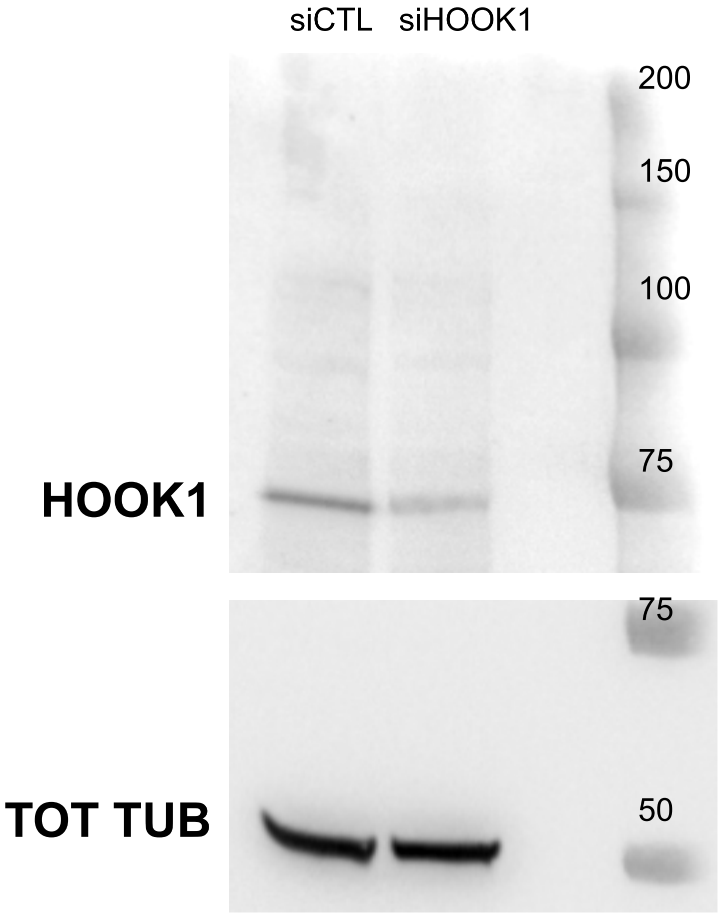

siRNA against HOOK1 mRNA was used after 72h of cell culture

Figure 7C (2)

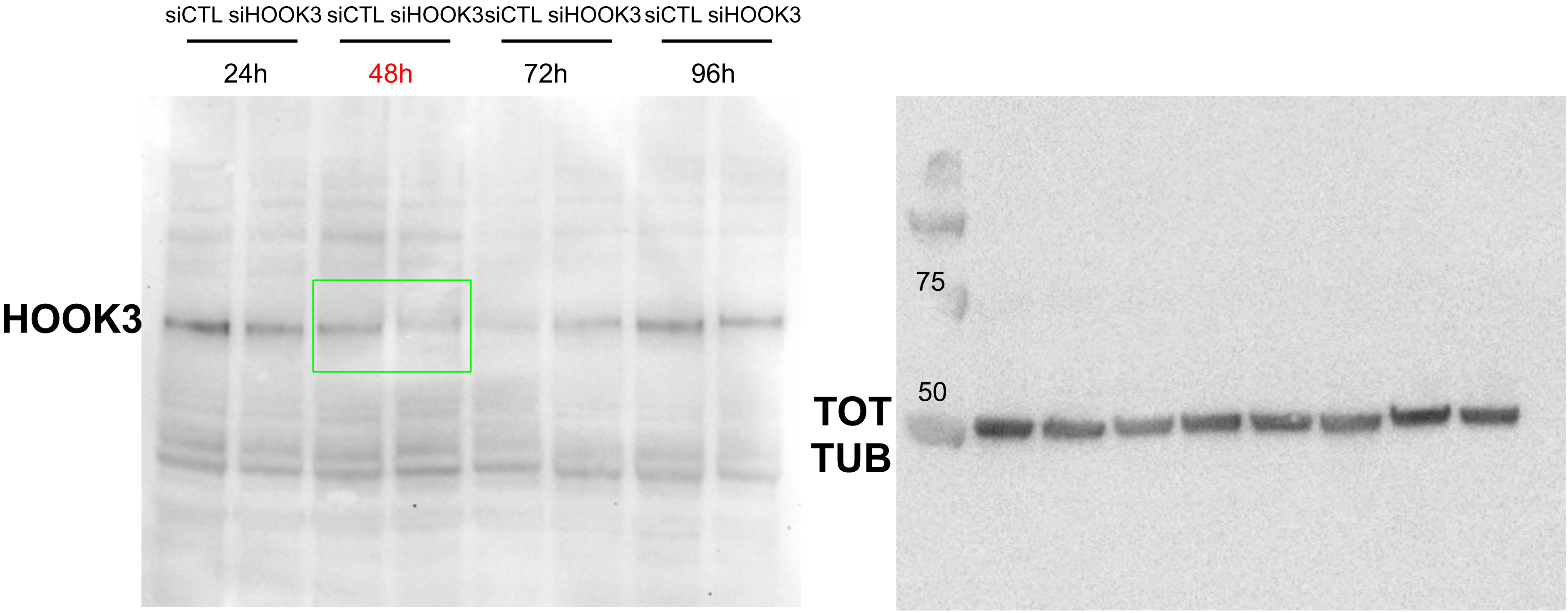

siRNA against HOOK3 mRNA was used after 48h of cell culture

Figure 7G

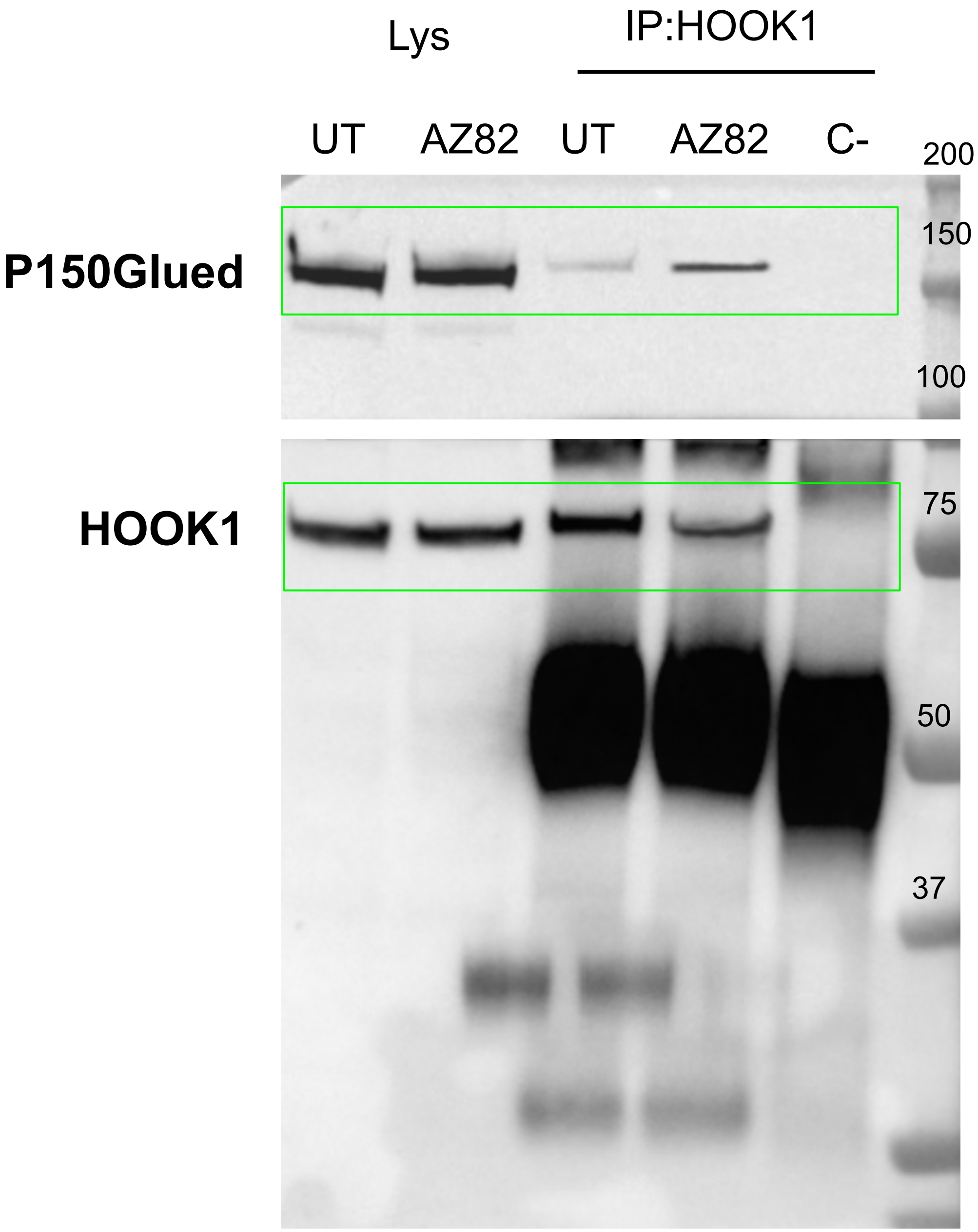

Lys
IP:HOOK1
UT
AZ82
UT
AZ82
C-
200
150
100
P150Glued
HOOK1
75
50
37
