# Supplementary material for: Distinct retrograde microtubule motor sets drive early and late endosome transport
Source: EMBO J. 2020 Nov 20;39(24):e103661. doi: 10.15252/embj.2019103661 (PMC7737607; doi:10.15252/embj.2019103661)
Supplement: Supplementary file 8 — Source Data for Expanded View [file EMBJ-39-e103661-s016.zip › Figure_EV2_Source_Data.zip!/Figure_EV2_Source_Data/Figure EV2_Source data.pdf]

Figure EV2E

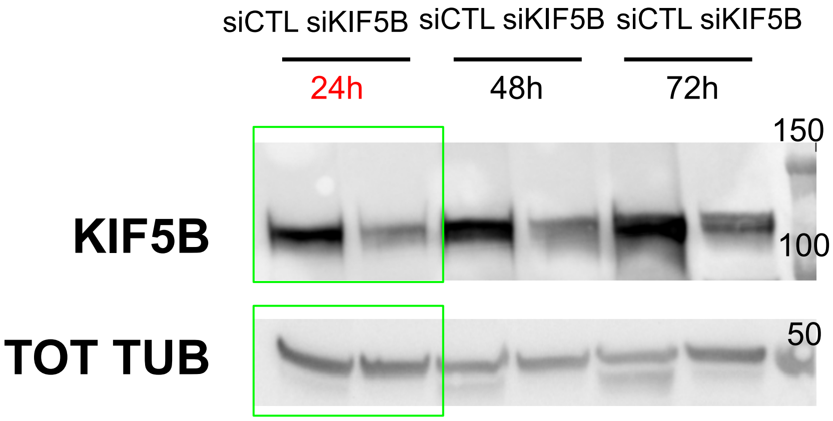

siRNA against KIF5B mRNA was used after 24h of cell culture
