# Supplementary material for: Distinct retrograde microtubule motor sets drive early and late endosome transport
Source: EMBO J. 2020 Nov 20;39(24):e103661. doi: 10.15252/embj.2019103661 (PMC7737607; doi:10.15252/embj.2019103661)
Supplement: Supplementary file 8 — Source Data for Expanded View [file EMBJ-39-e103661-s016.zip › Figure_EV3_Source_Data.zip!/Figure_EV3_Source_Data/FIgure EV3_Source data.pdf]

# Figure EV3A

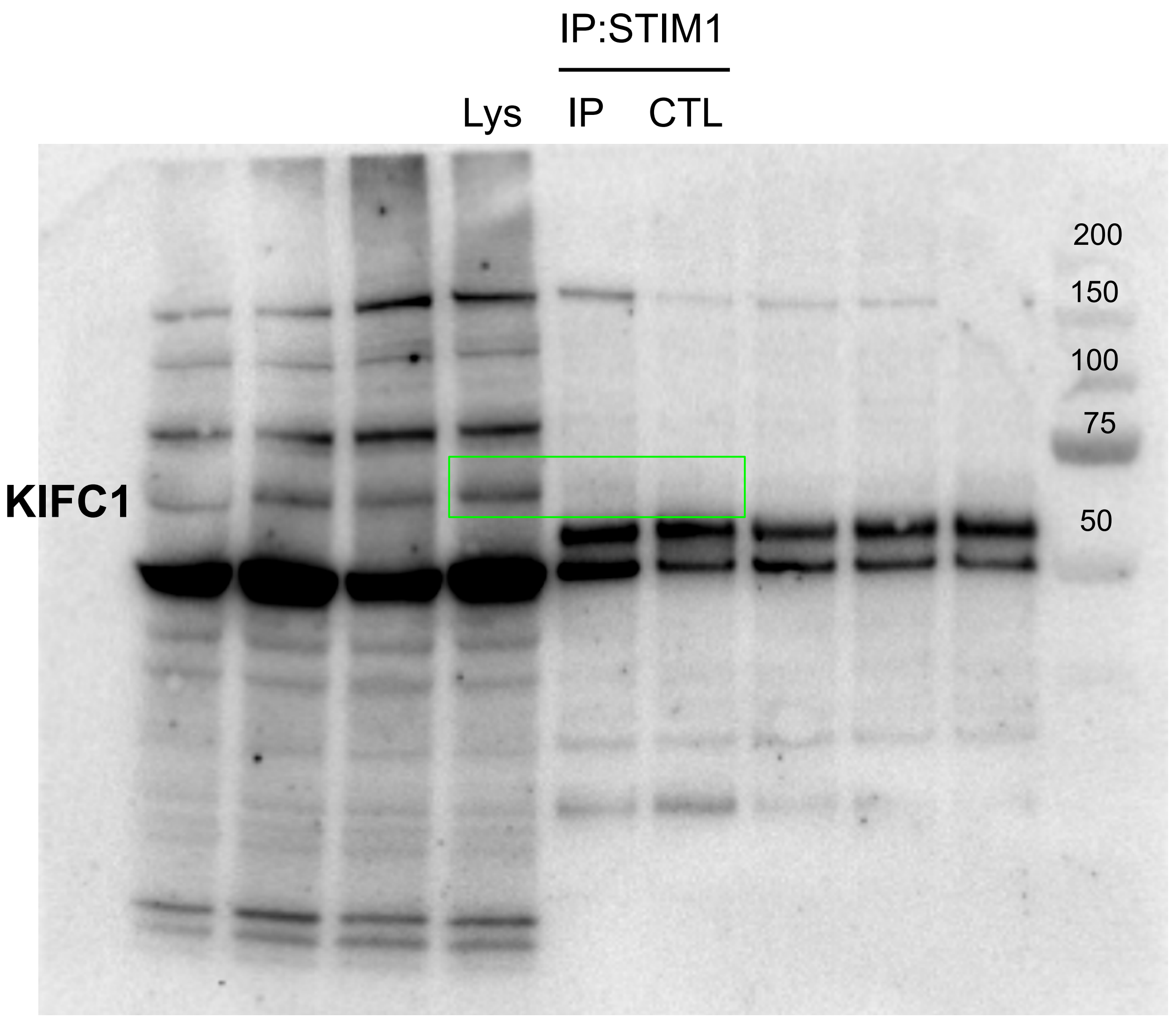

# Figure EV3A (2)_STRIPPED

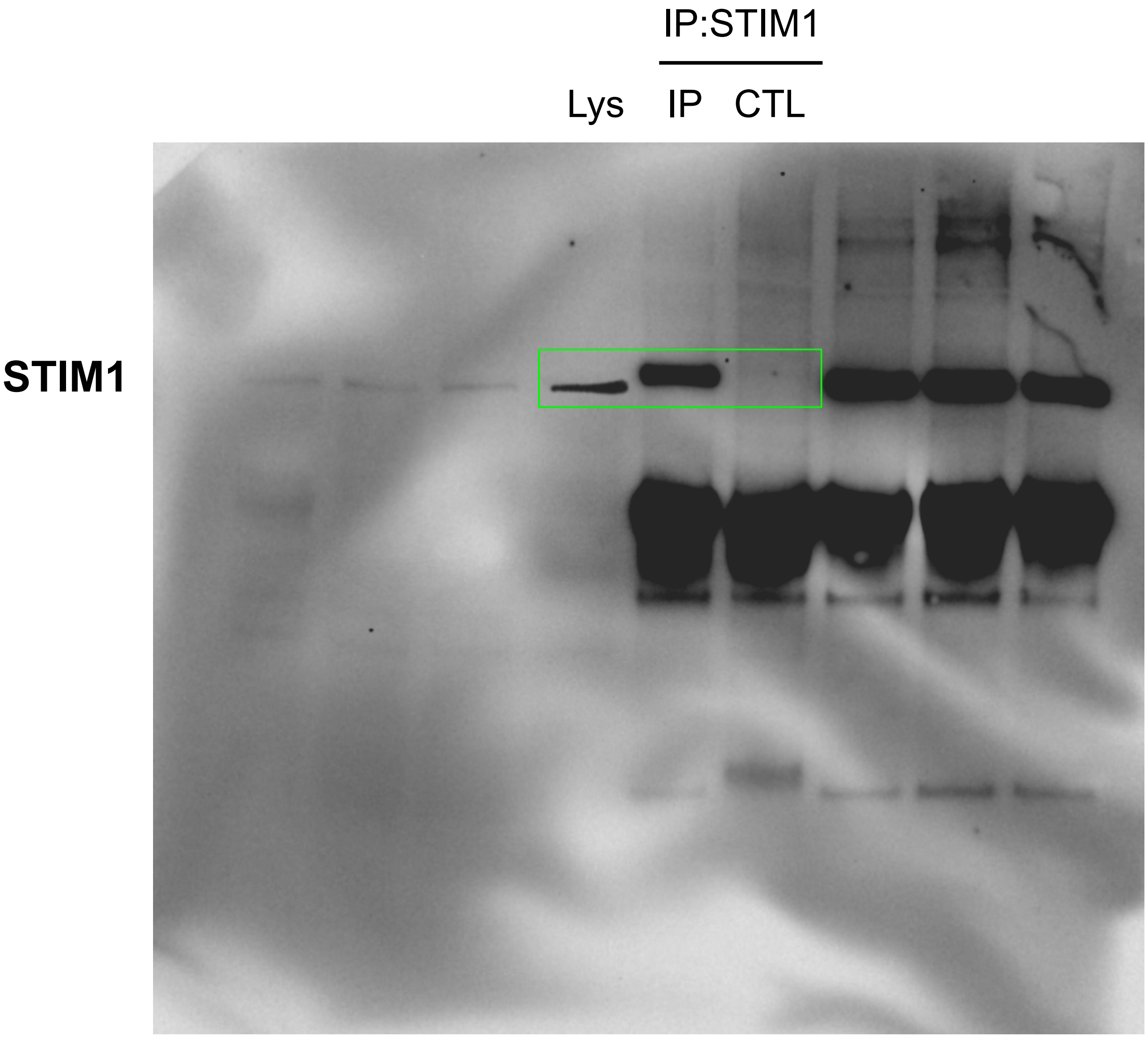

## Figure EV3D

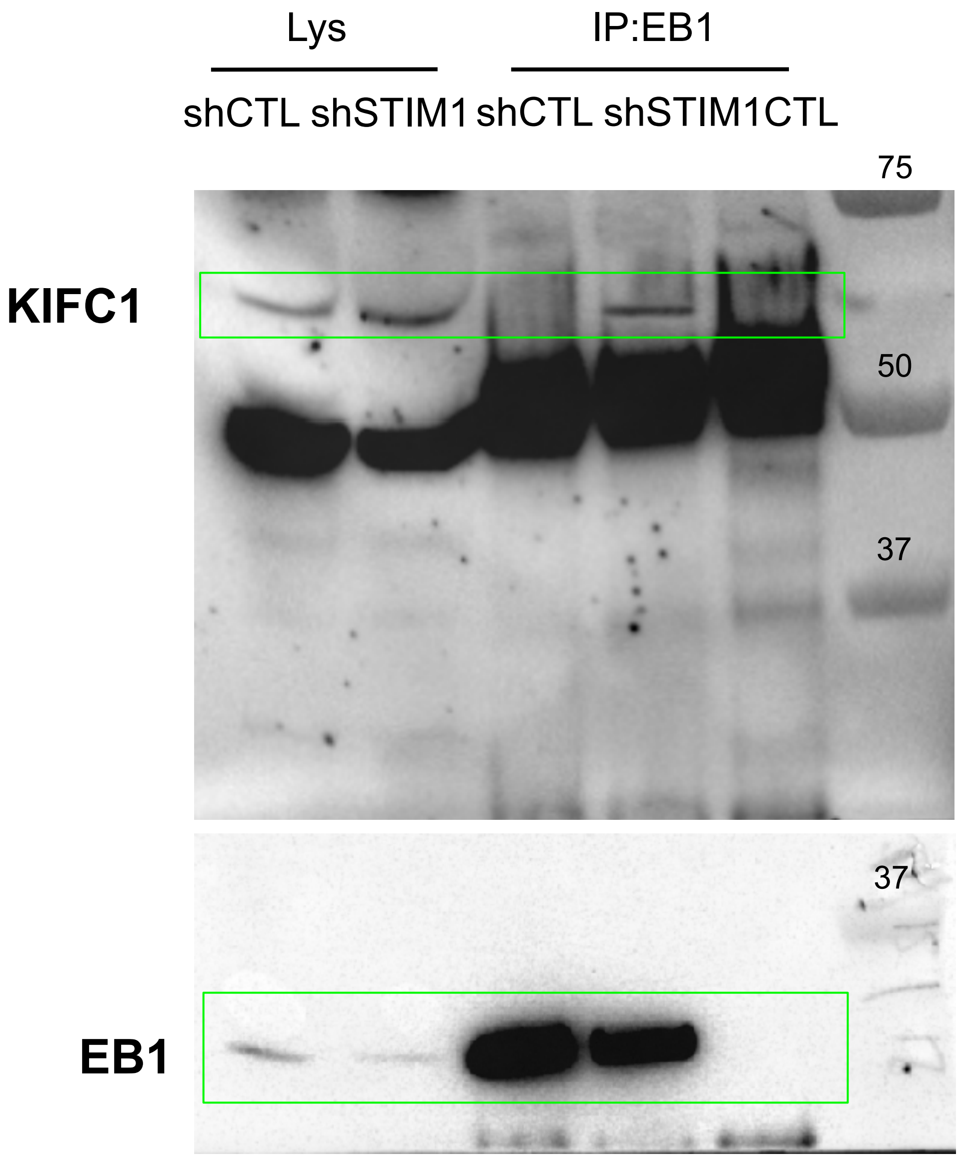
Lys
IP:EB1
shCTL shSTIM1 shCTL shSTIM1 CTL
75
50
37
KIFC1
37
EB1
