# Supplementary material for: Distinct retrograde microtubule motor sets drive early and late endosome transport
Source: EMBO J. 2020 Nov 20;39(24):e103661. doi: 10.15252/embj.2019103661 (PMC7737607; doi:10.15252/embj.2019103661)
Supplement: Supplementary file 8 — Source Data for Expanded View [file EMBJ-39-e103661-s016.zip › Figure_EV1_Source_Data.zip!/Figure_EV1_Source_Data/Figure EV1_Source data.pdf]

Figure EV1A

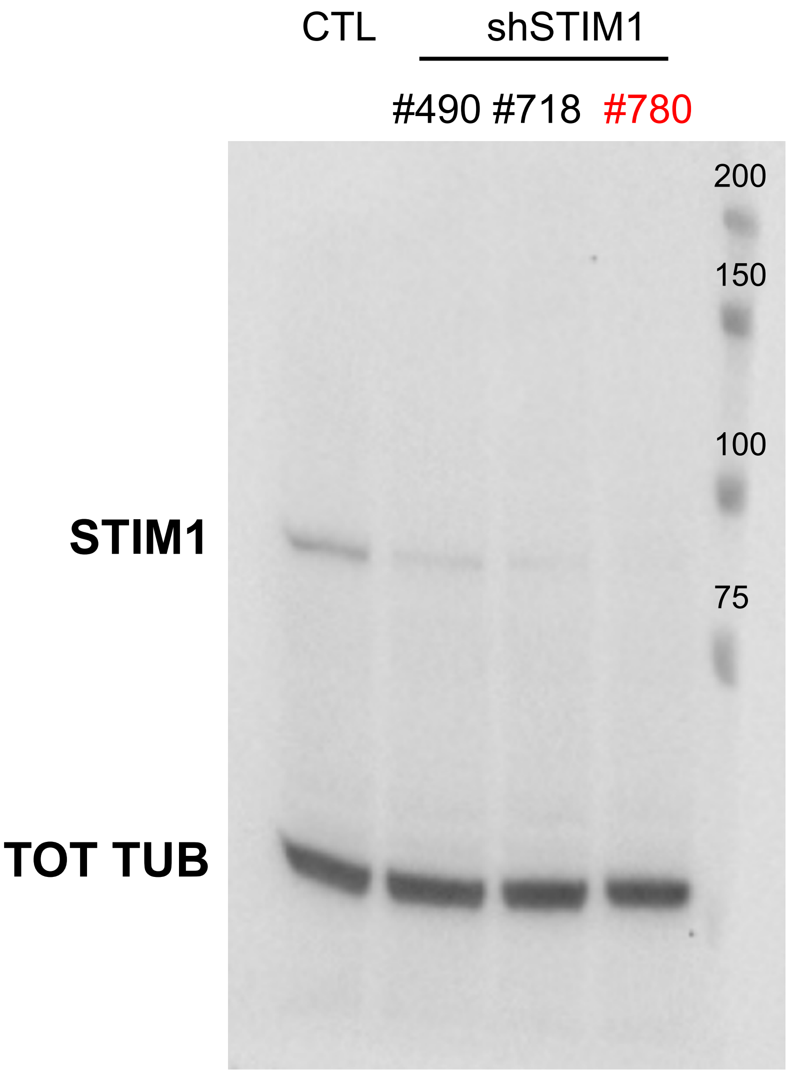

shRNA against STIM1 mRNA used is the #780

Figure EV1B

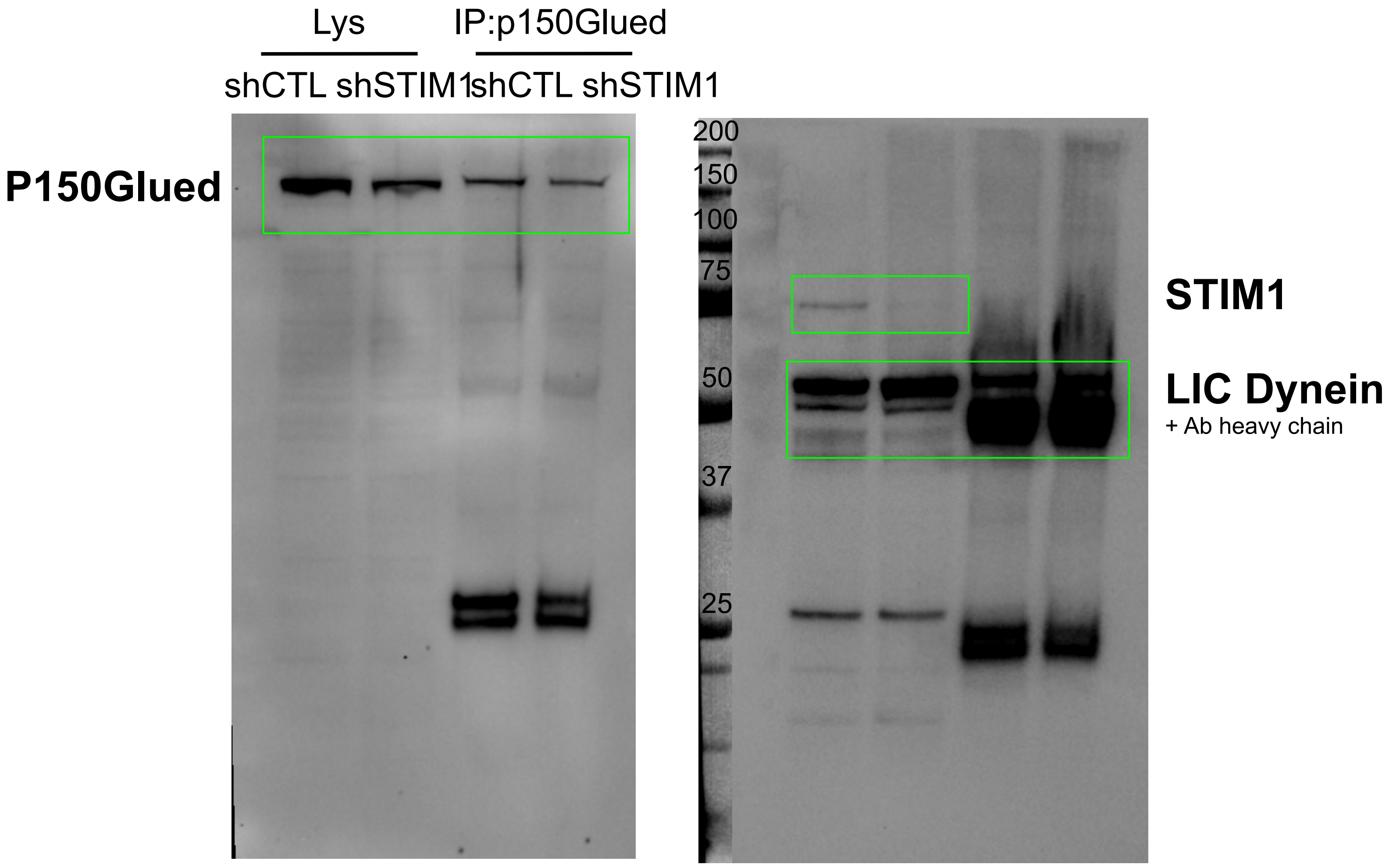

Lys
IP:p150Glued
shCTL shSTIM1 shCTL shSTIM1
P150Glued
200
150
100
75
50
37
25
STIM1
LIC Dynein
+ Ab heavy chain

Figure EV1C

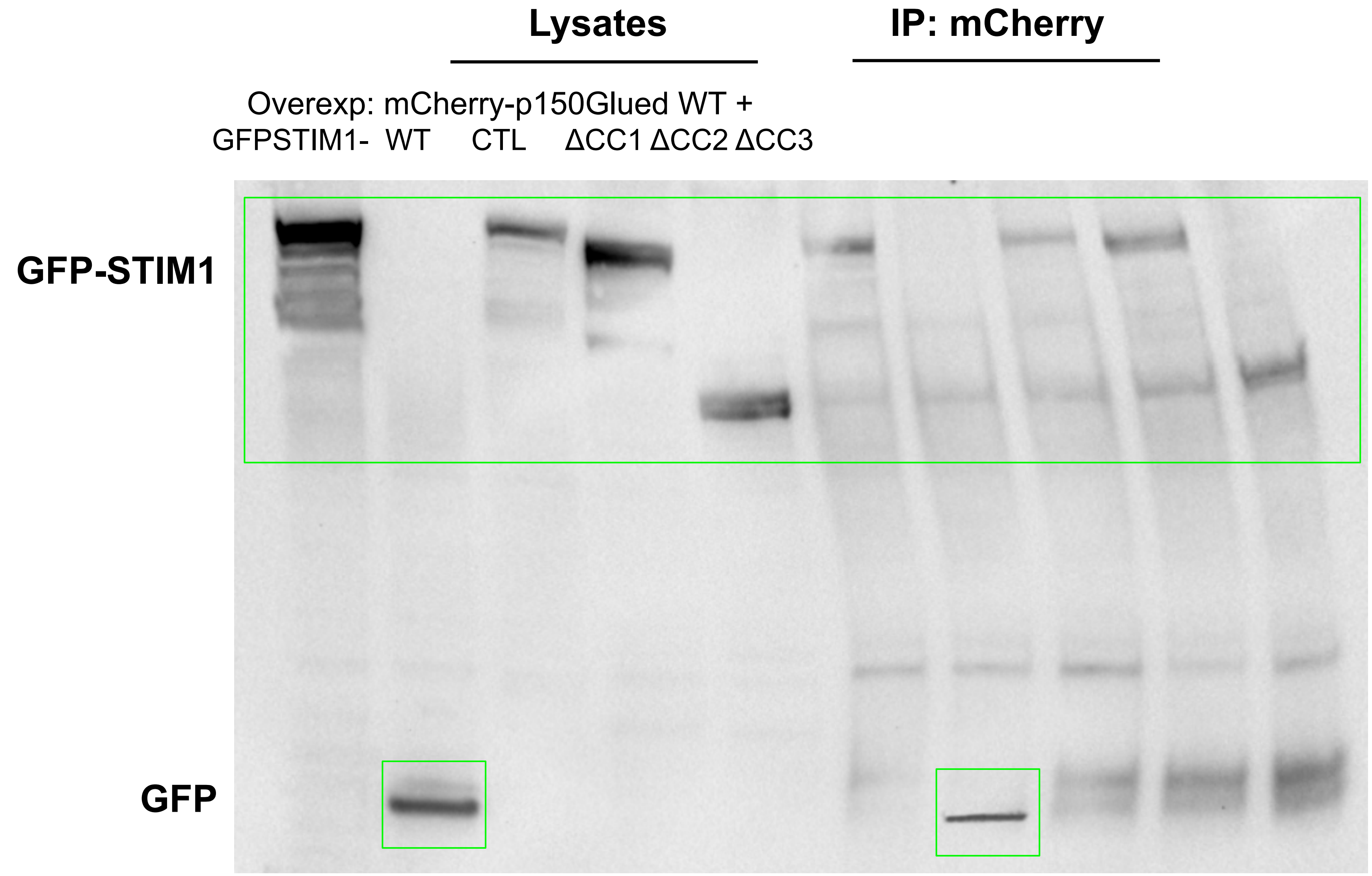

antiGFP used to detect GFP-tagged STIM1 constructs

Figure EV1C (2)_STRIPPED

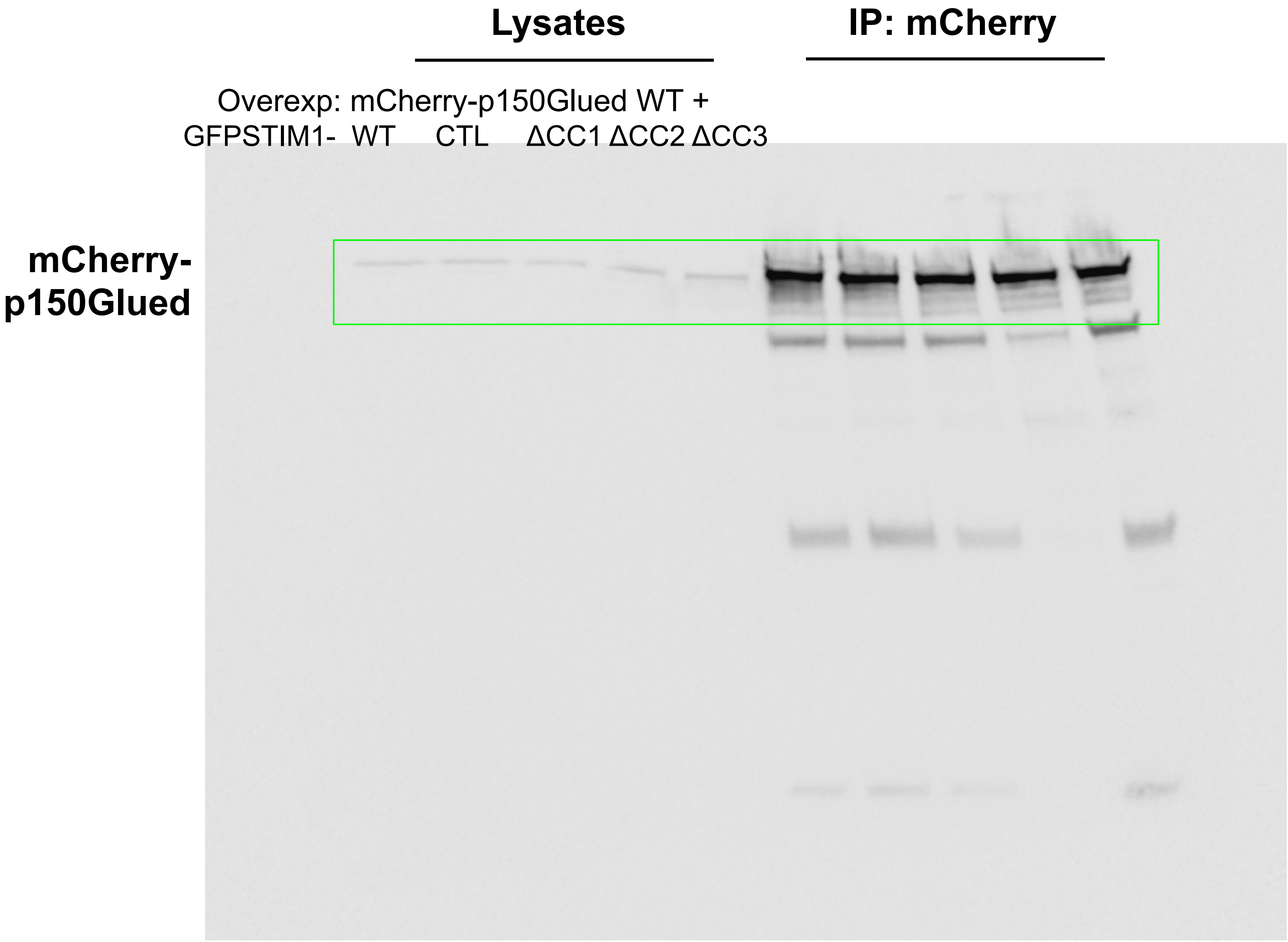

Anti-mCherry used to detect p150-Glued WT

Figure EV1D

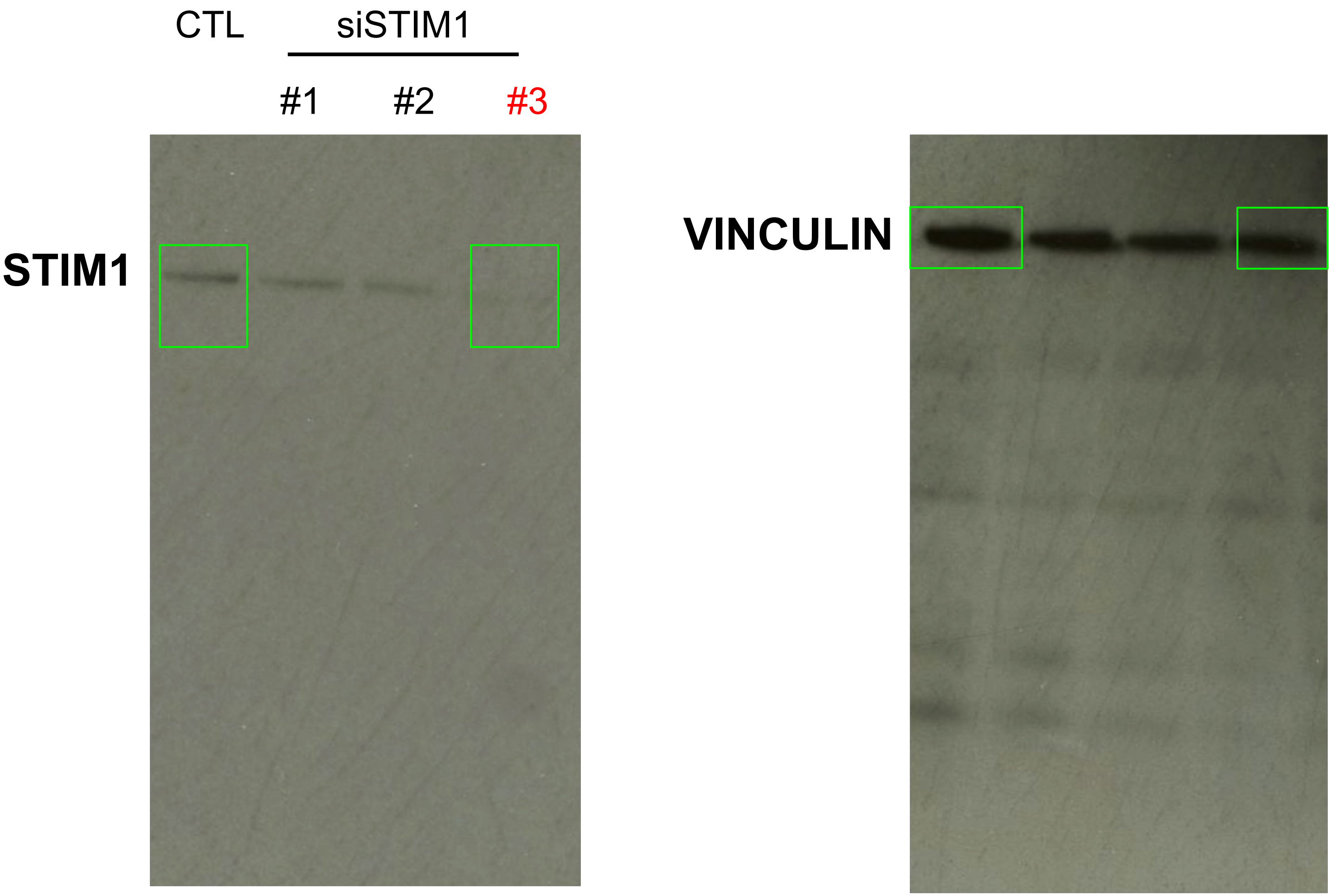

siRNA against STIM1 mRNA used is the #3 after 24h of cell culture
